# From anecdote to evidence: experimental validation of fire-cue recognition in Australian sleepy lizards

**DOI:** 10.1098/rsbl.2025.0364

**Published:** 2025-09-17

**Authors:** Chris J. Jolly, Dale G. Nimmo, Alexandra J. R. Carthey, Emma H. V. van de Pas, Martin J. Whiting

**Affiliations:** ^1^ School of Natural Sciences, Macquarie University, Sydney, New South Wales, Australia; ^2^ Research Institute for the Environment and Livelihoods, Charles Darwin University, Darwin, Northern Territory, Australia; ^3^ Gubali Institute, Charles Sturt University, Albury, New South Wales, Australia; ^4^ Department of Animal Management, Van Hall Larenstein University of Applied Sciences, Leeuwarden, Friesland, The Netherlands

**Keywords:** behavioural response, chemical cue, fire ecology, reptile, squamate, vomerolfaction

## Abstract

Fire has shaped the evolution of both plants and animals. Animals exposed to fire throughout their evolutionary history are predicted to exhibit behavioural adaptations that enhance survival during fire. Here, we investigated whether Australian sleepy lizards (*Tiliqua rugosa*), a large skink from fire-prone regions, recognize and respond to cues of fire. Motivated by reports of captive sleepy lizards reacting to smoke, we conducted behavioural trials exposing wild-caught sleepy lizards to the chemosensory (smoke) and auditory (fire sounds) cues of fire. Behavioural analysis revealed that sleepy lizards exhibited increased activity and significantly greater movements in response to smoke than to water vapour. They did not, however, react aversively to auditory cues of fire, suggesting a reliance on chemosensory rather than auditory cues for fire detection. Our findings provide empirical support for the hypothesis that chemosensory cues of fire elicit escape responses in animals from fire-prone regions, suggesting an evolved, likely innate, behavioural adaptation to recognize and respond to fire cues as indicative of a threat. As climate change increases the frequency and intensity of wildfires, understanding how animals perceive and respond to fire will prove crucial for predicting the threat posed by a more fire-prone future.

## 1. Introduction

When staff at Audubon Zoo accidentally burnt their lunch, they realized they were not alone in smelling the acrid smell of smoke. Australian sleepy lizards (*Tiliqua rugosus*) in the affected room began rapidly tongue-flicking, pacing and attempting to escape their enclosures. Notably, other captive reptile species from less fire-prone regions, and sleepy lizards in an unaffected room, did not respond. The keepers speculated that, although these lizards were mostly captive bred, their long evolutionary history with fire in their native range explained their strong behavioural response to its cues [1].

Fire has shaped life on Earth for hundreds of millions of years [2], exerting selective pressure on both plants [3] and animals [4–6]. Being immobile, plants have evolved well-documented structural and functional traits—such as fire-resistant bark and the ability to resprout after burning—that enhance their survival during fire and persistence in fire-prone regions [3]. By contrast, understanding and describing animal responses to fire—which are primarily behavioural—has proven more challenging [6]. Consequently, scientists have only recently begun systematically documenting how animals persist in fire-prone landscapes [7]. Yet, with Earth’s rapidly warming climate increasing the frequency and intensity of extreme wildfires [8], understanding how animals respond to fire has never been more urgent.

Anecdotes of animals responding to the cues of approaching fire—like the sleepy lizards at Audubon Zoo—prompted researchers to coalesce around the quest for a more unified understanding of these behavioural adaptations [4,7,9]. Animals that have evolved in fire-prone regions—such as sleepy lizards—are predicted to be able to detect the cues of fire, recognize the approaching, potentially fatal threat and mount a behavioural response to improve survival [6,7,9,10]. A sparse but growing body of literature suggests that some animals can indeed detect the chemosensory and auditory cues of incipient fire and respond accordingly [7]. In fire-prone regions of Australia, smoke’s chemosensory cues motivate eastern pygmy possums (*Cercartetus nanus*) and Gould’s long-eared bats (*Nyctophilus gouldi*) to rouse from torpor [11,12]. Mediterranean lizards (*Psammodromus algirus*) from fire-prone habitats react more strongly to the smell of smoke than do lizards from habitats where fire is rare [13]. Western fence lizards (*Sceloporus occidentalis*), chalky reed frogs (*Hyperolius nitidulus*) and eastern red bats (*Lasiurus borealis*) appear to recognize and respond to the sound of fire [14–16]. Yet, it remains unclear whether the salience of the different fire-cue modalities (i.e. chemosensory, auditory, visual, multimodal) is species- and context-dependent [7].

Here, we add to this emerging literature by revisiting the anecdotal observations reported by Mendyk *et al.* [1]. We experimentally test whether Australian sleepy lizards can detect and respond to cues associated with approaching fire. We presented wild-caught sleepy lizards with the chemosensory cues of fire (smoke), a potentially long-range signal of approaching fire [7], paired with a water vapour control. Additionally, we introduced auditory cues of fire (recordings of a wildfire), which provide a more imminent and localized signal of approaching fire [7], paired with a white noise control. For each cue, we quantified behavioural responses and measured their magnitude. If sleepy lizards recognize that fire-related stimuli predict a threat (direct mortality), we predict that they will attempt to flee when exposed to smoke and to fire sounds, but not to water vapour or white noise.

## 2. Methods

Australian sleepy lizards (*Tiliqua rugosa*), also known as shinglebacks or bobtails, are a large (mean snout–vent length approx. 30 cm [17]), viviparous, terrestrial skink, with an omnivorous diet [18]. They are widely distributed across southern Australia and inhabit a variety of mesic to arid habitats, including coastal heaths, eucalypt woodlands, *Acacia* scrublands and spinifex-dominated deserts [18,19]. Sleepy lizards typically shelter under shrubs and ground debris, such as logs and sheets of iron, but will seek shelter in the burrows of other animals during extreme temperatures [20]. Sleepy lizards are long-lived (at least 50 years [21]), experience low adult mortality [21], form monogamous breeding pairs [22] and produce only a few large offspring per litter (1–3 neonates [23]), which take 3−4 years to reach sexual maturity [21].

In 2018, 10 adult female sleepy lizards were collected from near Lake Victoria (−34.0, 141.3), southwest New South Wales, Australia, as part of ongoing research at Macquarie University (e.g. [24–26]). Lake Victoria is in the Murray–Darling Depression bioregion, which experiences a warm arid climate and is dominated by mallee shrublands [27]. This region experiences medium-intensity shrub fires in spring and summer with historical fire return intervals of 20−100 years [28]. Following collection, the lizards were housed at Macquarie University’s animal husbandry facility in temperature-controlled rooms and, during periods of low humidity, occasionally outdoors. All lizards were held in captivity from 2018 until the commencement of this study between September and November 2023. During this time, individuals were housed separately in open-topped plastic tubs (80 L × 60 W × 45 H cm) and individually marked for identification.

To test the behavioural response of sleepy lizards to the chemosensory and auditory cues of fire, we exposed each lizard to four treatments: a chemosensory cue (smoke), a control (water vapour), an auditory cue (recording of a wildfire) and an auditory control (white noise). Each lizard received all four treatments on three separate occasions, resulting in a total of 12 trials per individual. Trials were conducted in each lizard’s home enclosure, with all furnishings (i.e. water and food bowls, logs), except for a substrate of wood shavings, removed. All enclosures were fitted with a clear acrylic lid to which a camera was mounted. All auditory trials were conducted in a temperature-controlled (mean trial temperature ± SE = 31.34 ± 0.1°C; mean trial humidity ± SE = 39.15 ± 0.65%), relatively soundproof experimental room. To avoid setting off smoke alarms, all chemosensory trials were conducted outdoors under the shelter of a veranda awning, which prevented direct sun exposure and helped maintain consistent conditions across trials. Outdoor chemosensory trials were conducted on days predicted to be ≥30°C and at the same time of day (mean trial temperature ± SE = 30.5 ± 0.37°C; mean trial humidity ± SE = 27.86 ± 1.41%). Across all trials, humidity and temperature (°C) were recorded immediately prior to each trial.

Prior to each trial, lizards in their enclosures were placed in the experimental room (auditory trials) or outside (chemosensory trials) and were allowed to acclimatize for 1 h. After the acclimatization period, lizard pre-trial behaviour was recorded for 10 min using a GoPro Hero7 Silver (GoPro Inc., San Mateo, California, USA) mounted above the centre of each enclosure. After the pre-trial period, all lizards were exposed to one of the four treatments, and their behaviour was recorded for a further 10 minutes.

To expose lizards to smoke, we burnt dry native grass (*Heteropogon contortus*) through an infusion smoker (Davis and Waddell, Preston, Queensland, Australia). Smoke was blown for 5 s through a rubber tube directly into each enclosure via an opening on the top of a wall of the enclosure. To expose lizards to water vapour, we used a Hurricane 700 fog machine (Chauvet DJ, Sunrise, Florida, USA) to blow odourless water vapour for 5 s through a rubber tube using the same method as the treatment. This methodology attempted to expose lizards to similar visual cues, but only smoke provided the chemical cues of fire [13].

To expose lizards to the auditory cues of fire, we played a recording of a wildfire from Álvarez-Ruiz *et al.* [16]. As an auditory control, we exposed lizards to a recording of white noise from Bent *et al.* [29]. Auditory treatments were played through a WONDERBOOM Portable Bluetooth Speaker (Ultimate Ears, Irvine, California, USA) at full volume. The speaker was positioned in the middle of the room approx. 1 m above the ground and approx. 1 m from each lizard enclosure. We recorded the maximum decibels and average decibels of the recordings during each trial.

The order in which individuals were exposed to treatments (smoke or the sound of fire) and controls (water vapour or white noise) was randomized; however, auditory trials were typically conducted first, and the chemosensory trials were conducted when outside temperature allowed. After every individual had been exposed to all four treatments, the treatments were repeated twice more (i.e. each lizard was exposed to the four treatments three times).

We assessed lizard behavioural responses to the cues of fire by autonomously tracking their movement during the 10 min trial period using the video tracking software AnimalTA, v. 3.1.0 [30]. AnimalTA tracked the proportion of time spent moving and distance travelled (cm) by each lizard before and after exposure to treatments. Videos were tracked at a frame rate of 7.5 frames s^−1^ and the moving threshold was set to 0.2 mm s^−1^. This moving threshold detected stationary movements (i.e. walking on the spot against enclosure walls) without excessive detection sensitivity. Because lizards use their tongue to detect chemosensory cues, we also used BORIS v. 8.22.6 [31] to score the number of times each lizard extended and retracted its tongue during each trial. All tongue-flicks were counted by a single observer (EHVvdP), who was blinded to the treatment being scored where possible (i.e. with sound muted). Despite precautions, in some cases smoke and vapour could be differentiated.

All statistical analyses were performed using R v. 4.3.1 [32]. To test the behavioural response of sleepy lizards to the chemosensory and auditory cues of fire, we ran generalized linear mixed models (GLMMs), fitted via the *glmmTMB* package [33], to compare the behavioural responses of lizards (i.e. proportion of time spent moving, distance travelled and tongue-flick count) between treatments (smoke or fire sounds) and controls (water vapour or white noise). Proportion of time spent moving was modelled using GLMMs with a beta distribution and logit link function. Because the beta distribution requires values strictly between 0 and 1, we applied a bias correction transformation to this response variable. Distance travelled by lizards was analysed using GLMMs with a gamma distribution after adding 0.01 to the response variable to avoid zeros. Tongue-flicking behaviour was modelled using negative binomial GLMMs to account for overdispersion. In all models, we initially included interactive effects of treatment, temperature, humidity and—for auditory trials only—average decibel level. No interactions were significant. We then used Akaike’s information criterion (AIC) to compare models with and without non-significant fixed effects and retained models with the lowest AIC values. Ultimately, all fixed effects except for the treatment variable were excluded, as they did not improve model fit. All models included a random intercept for individual lizard to account for repeated trials.

## 3. Results

**Figure 1.**
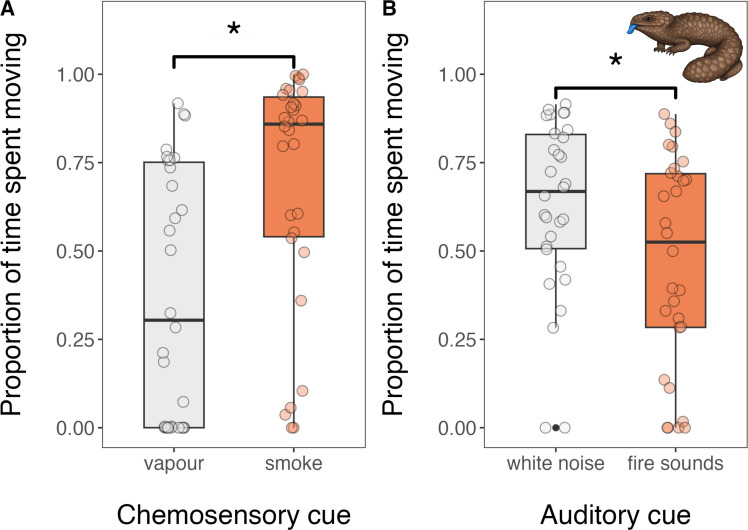
The proportion of time Australian sleepy lizards (*Tiliqua rugosa*) spent moving after exposure to the chemosensory and auditory cues of fire paired with controls during a 10 min behavioural trial. Statistically significant effects (*p* < 0.05) are denoted by an asterisk.

**Figure 2.**
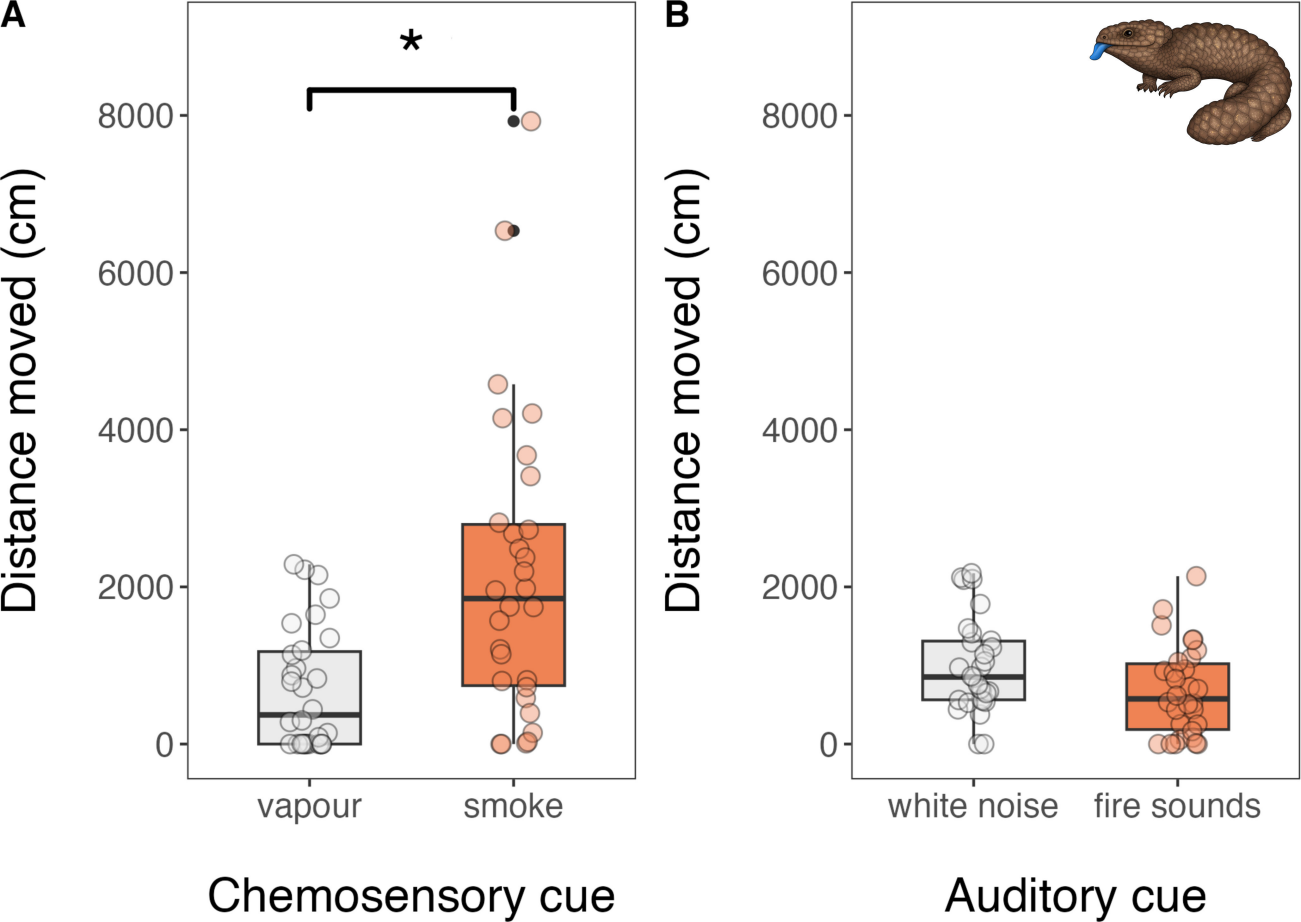
The distance travelled (cm) by Australian sleepy lizards (*Tiliqua rugosa*) following exposure to the chemosensory and auditory cues of fire paired with controls during a 10 min behavioural trial. Statistically significant effects (*p* < 0.05) are denoted by an asterisk.

**Figure 3.**
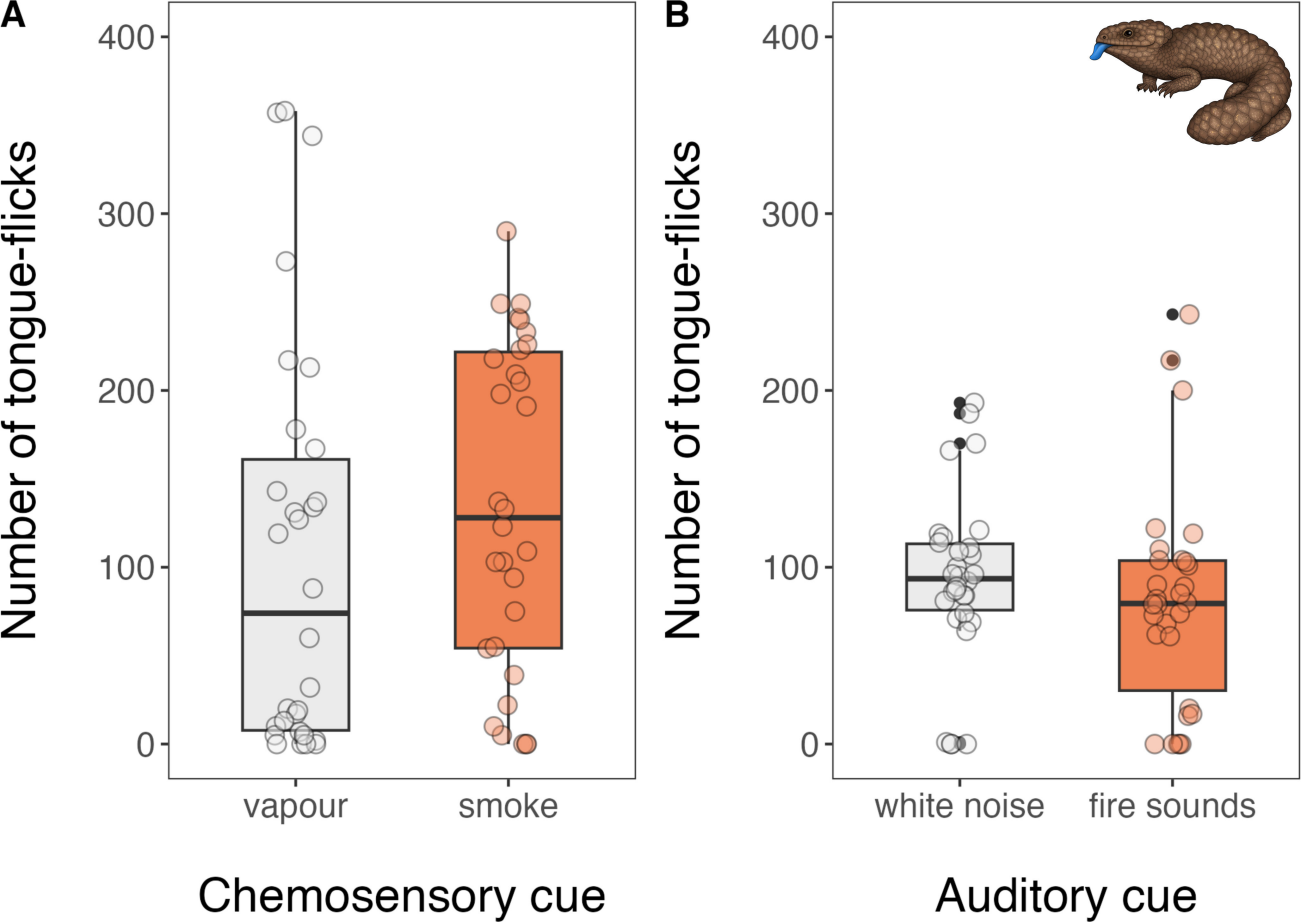
The number of times Australian sleepy lizards (*Tiliqua rugosa*) flicked their tongue following exposure to the chemosensory and auditory cues of fire paired with controls during a 10 min behavioural trial.

Following exposure to smoke, sleepy lizards spent a significantly higher proportion of their time moving (x̄ ± SE = 0.69 ± 0.06) than when they were exposed to vapour (x̄ ± SE = 0.38 ± 0.06; *β* = −1.26, 95% CI = [−1.88, −0.64], *p* < 0.001; figure 1). Sleepy lizards also travelled (fled) significantly further when exposed to smoke (x̄ ± SE = 2153 ± 352 cm) than when they were exposed to vapour (x̄ ± SE = 693 ± 141 cm; *β* = −1.14, 95% CI = [−2.11, −0.17], *p* = 0.022; figure 2). When exposed to the sound of a wildfire, sleepy lizards spent a significantly lower proportion of their time moving (x̄ ± SE = 0.47 ± 0.05) than when they were exposed to white noise (x̄ ± SE = 0.63 ± 0.05; *β* = 0.66, 95% CI = [0.14, 1.19], *p* = 0.013; figure 1). However, there was no significant difference in distance travelled (fled) between white noise (x̄ ± SE = 955 ± 110 cm) and the sound of fire (x̄ ± SE = 675 ± 103 cm; *β* = 0.39, 95% CI = [−0.33, 1.11], *p* = 0.288; figure 2). Neither chemosensory (smoke: x̄ ± SE = 134.5 ± 17.0 tongue-flicks; vapour: x̄ ± SE = 105.9 ± 21.0 tongue-flicks; *β* = −0.50, 95% CI = [−1.19, 0.19], *p* = 0.156) nor auditory (burning: x̄ ± SE = 79.9 ± 11.3 tongue-flicks; white noise: x̄ ± SE = 92.8 ± 9.0 tongue-flicks; *β* = 0.19, 95% CI = [−0.20, 0.58], *p* = 0.346) fire cues affected tongue-flicking behaviour (figure 3).

## 4. Discussion

Our findings support the hypothesis that Australian sleepy lizards (*T. rugosa*) can detect the chemosensory cues of fire—specifically smoke—and respond with an appropriate escape behaviour. This provides empirical support for recent anecdotal observations [1] and strengthens a growing body of evidence that animals from fire-prone ecosystems recognize smoke as heralding imminent fire and mount a behavioural response to avoid it (e.g. [11–13]). Importantly, the lack of comparable response to the auditory cues of fire—despite increased movement in response to white noise—suggests that these lizards may rely primarily on chemosensory cues for fire detection. Despite this, and perhaps surprisingly, sleepy lizards flicked their tongues (i.e. sampling airborne chemical cues) no more frequently in response to smoke than to vapour.

Most ecosystems inhabited by sleepy lizards are fire-adapted, with return intervals typically ranging from 20 to 100 years [28]. Despite the long lifespan of sleepy lizards (approx. 50 years [21]), some individuals may never experience fire. Because our study lizards were captured as adults, it is unclear whether they had any prior exposure to fire. However, their capture site (Lake Victoria, NSW) has not experienced fire in over 50 years [34], suggesting it has not burnt within the lifetime of most sleepy lizards. Similarly, many of the sleepy lizards that responded to the burnt lunch at Audubon Zoo were captive-born and would have had no exposure to fire. Thus, recognizing smoke as a signal of imminent fire and fleeing in response likely represents an evolved, innate behaviour [1,7]. Innate recognition of smoke as heralding fire suggests that similar behavioural responses may be widespread among animals that co-evolved with fire, providing an evolutionary advantage by reducing fire-related mortality [7,35].

Squamate reptiles possess sophisticated chemosensory organs evolved to detect and discriminate the chemical signatures of foods, predators and conspecifics [36]. The evolutionary pressure exerted by fire may have allowed reptiles to co-opt these sensory adaptations, enabling them to recognize and respond to chemosensory cues associated with fire. Fire has been a recurring threat throughout the evolutionary history of many species, particularly in fire-prone regions such as Australia, where natural fires are common and often destructive [2]. This persistent environmental disturbance likely drives selective pressures favouring individuals that can effectively perceive and respond to fire cues [4,6,7]. Our findings align with recent studies of other reptiles, such as the Mediterranean lizard (*P. algirus*), which exhibits stronger responses to smoke when sourced from fire-prone areas [13]. Such convergent adaptations across species and continents suggest that the ability to detect fire cues may be widespread among reptiles in fire-prone regions.

While our findings support the idea that fire has shaped responses to smoke in sleepy lizards, our interpretation is somewhat limited by our lack of inter- and intra-specific comparisons. We did not test whether closely related species—or sleepy lizard populations from regions with differing fire regimes—respond differently to fire cues but acknowledge such comparisons would help disentangle whether fire responsiveness reflects evolutionary adaptation versus more generalized threat detection. However, while sleepy lizards are widespread, they typically occupy habitats with broadly similar fire return intervals [18,28]. This reduces the likelihood of major differences in fire exposure among populations. Moreover, their long lifespans and large home ranges [18] make it difficult to determine how many fire events any individual—or population—has experienced over ecological or evolutionary timescales.

Interestingly, the response of sleepy lizards to smoke is analogous to how prey animals recognize and respond to predator cues. Much like the imminent threat of a predator, fire poses a potential for immediate harm or even death, motivating animals to rapidly mount an effective escape response [37,38]. In both scenarios, the perception of the threat—whether from a predator or fire—initiates a behavioural response that increases the animal’s chances of survival. For example, mammals and birds can detect visual, auditory and olfactory cues of predators to assess and mitigate the risk of predation [39–41]. Similarly, sleepy lizards’ heightened activity levels and increased distance travelled upon detecting smoke indicate that their reaction is not unlike appropriate antipredator behaviour [7].

Our findings that sleepy lizards responded to chemosensory but not auditory cues of fire may reflect the varying reliability of these sensory inputs for survival decisions in reptiles [7]. The smell of smoke is universally associated with fire, providing a highly salient and reliable cue for impending danger [42]. Interestingly, the frequency of tongue-flicking by sleepy lizard did not differ between smoke and vapour treatments. Potentially, the strong chemical signature of smoke may be detected with as little as a single tongue-flick, eliminating the need for additional information-gathering before enacting a behavioural response. In contrast, the sound of fire may be a less reliable cue to the level of danger posed by fire, particularly for reptiles with limited auditory sensitivity [43]. Additionally, the lizards’ reaction to white noise in the auditory trials—increased movement without corresponding increases in distance travelled—may reflect general arousal to a loud, novel sound rather than a targeted escape response to a cue associated with danger. This interpretation suggests that sleepy lizards do not associate the sound of fire with the same urgency as smoke, underscoring the importance of specific sensory mechanisms adapted to process threat-specific cues. In species where chemosensory sense organs are well-developed and chemosensory cues may be more informative, the use of auditory cues to gather information about threats might be attenuated or absent altogether.

The prioritization of chemosensory cues over auditory cues may also stem from phylogenetic, ecological and behavioural constraints. In open landscapes, like those inhabited by sleepy lizards, smoke can travel over considerable distances, offering an early warning system for approaching fire [7,44]. In contrast, sound dissipation in open habitats and the lower auditory acuity of non-vocal reptiles compared to mammals and birds [45] may reduce the selective advantage of responding to the sound of fire. It is worth noting, however, that western fence lizards [16], chalky reed frogs [14] and eastern red bats [15] have all been documented to respond to the sound of fire, so clearly it can be a salient cue in certain habitats and species, but perhaps not all. Thus, the absence of a strong response to auditory cues may reflect a habitat- and species-specific sensory prioritization, with sleepy lizards evolving heightened chemosensory sensitivity to smoke rather than auditory sensitivity to fire sounds.

Understanding the sensory mechanisms and behavioural responses of animals to the cues of fire has significant conservation implications, particularly given the increasing frequency and intensity of wildfires driven by climate change [2,8] and their impacts on biodiversity [46]. Animals that rely on fire cues for survival may experience elevated mortality in fires exceeding historical intensities and/or frequencies, especially if they adopt a survival strategy that is inappropriate or insufficient for novel fire conditions (see [7]). Additionally, as human activities increase, habitat fragmentation and altered fire regimes may disrupt the natural conditions in which these behaviours evolved, potentially compromising the effectiveness of adaptive responses.

Our findings demonstrate that Australian sleepy lizards recognize and respond to chemosensory fire cues [1], underscoring the role of fire as an evolutionary force shaping behavioural adaptations in animals from fire-prone regions. This study adds to a growing body of evidence that animals exposed to the selective pressure of fire evolve unique strategies to evade its lethal consequences [4,6,7]. These findings highlight the need for continued research into fire-driven selection across taxa and habitats to better understand how animals may cope with rapidly changing fire regimes in an era of global climate change. Future studies comparing the fire-cue responsiveness of closely related species or populations that vary in their evolutionary exposure to fire would be especially valuable for disentangling the role of selection in shaping these behaviours.

## Data Availability

All data and code are available from the Dryad Digital Repository [47].
